# Gram-negative bacterial sepsis, antimicrobial susceptibility pattern and treatment outcomes at two neonatal intensive care units in Addis Ababa, Ethiopia: A retrospective observational study

**DOI:** 10.1371/journal.pone.0323288

**Published:** 2025-05-13

**Authors:** Biniyam Tedla Mamo, Zelalem Tazu Bonger, Feyissa Regassa Senbato, Tadesse Eguale, Kibrewossen Kiflu Akililu, Samuel Muluye Welelaw, Eden Dagnachew Zeleke, Asrat Demtse, Turegne Assefa, Ruth Woldeyohannes Yirgu, Zelalem Mekuria, Joan-Miquel Balada-Llasat, Shu-Hua Wang

**Affiliations:** 1 Ohio State Global One Health, LLC, Addis Ababa, Ethiopia; 2 Department of Pediatrics and Child Health, School of Medicine, College of health Sciences, Addis Ababa University, Addis Ababa, Ethiopia; 3 Department of Pediatrics and Child Health, Zewditu Memorial Hospital, Addis Ababa, Ethiopia; 4 The Ohio State University, College of Medicine, Global One Health Initiative, Columbus, Ohio, United States of America; 5 The Ohio State University, College of Veterinary Medicine, Columbus, Ohio, United States of America; 6 Department of Pathology, The Ohio State University, College of Medicine, Columbus, Ohio, United States of America; 7 Department of Internal Medicine, Division of Infectious Disease, The Ohio State University, College of Medicine, Columbus, Ohio, United States of America; Debre Markos University, ETHIOPIA

## Abstract

**Background:**

Neonatal sepsis is a leading cause of mortality and morbidity. To improve the clinical outcomes of neonates with sepsis, treatment should be based on bacteriological identification and antibiotic susceptibility. This study aims to assess the proportion of culture-positive gram-negative bacteria (GNB), the antibiotic susceptibility patterns, and treatment outcomes of neonatal sepsis at two neonatal intensive care units (NICUs) in Addis Ababa.

**Methods:**

A retrospective observational study was conducted among gram-negative sepsis suspected neonates admitted at Zewditu Memorial Hospital and Tikur Anbessa Specialized Hospital NICUs from January to December 2023. All neonates who were suspected of having sepsis were included in this study. Standard microbiological culture and biochemical tests were used to identify bacterial species and the Kirby-Bauer disc diffusion assay using Mueller-Hinton agar was employed to test the antimicrobial susceptibility of bacterial isolates as per Clinical Laboratory Standard Institute guidelines. Descriptive statistics were used to describe the study variables. Bivariable and multivariable logistic regression analyses were used to identify the factors associated with the treatment outcomes of neonatal sepsis. A p-value < 0.05 was set for statistical significance.

**Results:**

A total of 933 neonates were diagnosed with sepsis during the study period, of which 166 neonates were enrolled in the study for gram-negative sepsis: 84 (51%) were female and 97 (58%) had early onset sepsis. The median length of hospital stay was nine days with interquartile range of 16 days. The predominant GNB identified was *Klebsiella spp.* (n = 89; 49%), followed by *Acinetobacter spp*. (n = 38; 21%) and *Escherichia coli* (n = 19; 11%). In both hospitals, *Klebsiella spp.* was resistant to most of the routinely prescribed antibiotics: (n = 68; 89%) were resistant to ceftriaxone, (n = 56, 89%) cefepime and (n = 60; 75%) to gentamicin. Lower rates of resistance were recorded for other antibiotics such as ciprofloxacin (n = 12; 18%), ertapenem (n = 11; 16%), meropenem (n = 9; 13%), and amikacin (n = 3; 4%). A total of 92 (55%) neonates with the GNB isolated in the current study had multidrug-resistant (MDR) organisms. The study found that newborns with MDR infections were five times more likely to experience poor treatment outcomes compared to those with non-resistant strains (AOR, 5.23 95% CI [2.59, 11.11]). In addition, newborns who stayed less than seven days, compared to those who spent seven or more days in the hospital was four times (AOR: 4.16, 95% CI (2.0–9.01) more likely to experience poor health outcomes.

**Conclusion:**

*Klebsiella spp.* was the most common GNB isolated from the NICUs. More than half neonatal sepsis was caused by MDR organisms and associated with significant poor treatment outcomes. high prevalence of MDR-gram-negative bacteremia is alarming and highlights the need for the implementation of routine surveillance and infection control measures to decrease morbidity and mortality and to combat the development of antimicrobial resistance.

## Background

Globally, antimicrobial resistance (AMR) has become a major twenty-first century’s public health concern [1]. Estimating the burden of AMR worldwide is challenging because it requires high quality patient level clinical and laboratory data [2]. According to the World Health Organization (WHO), an estimated 1.05 million deaths associated with bacterial AMR were reported in the Africa region in 2019 [3]. Although, Ethiopia has limited data on AMR, some studies have shown a high prevalence of AMR in the country [4]. Recently, Ethiopia has been actively participating in the WHO’s Global Antimicrobial Resistance and Surveillance System (GLASS) by submitting antimicrobial susceptibility surveillance data from selected sentinel laboratories into the WHONET, a computerized microbiology data management and analysis program [5].

Sepsis is a leading cause of neonatal mortality and morbidity [6]. Globally, the number of reported instances of neonatal sepsis rose by around 12.8% between 1990 and 2019, an increase from 5.59 million to 6.31 million cases. Conversely, during the same timeframe, fatalities linked to neonatal sepsis declined from 0.26 to 0.23 million [6,7]. Previous studies in Africa reported that neonatal infections accounted for a quarter of the annual morbidity and mortality in neonates [8]. A recent meta-analysis conducted in Ethiopia showed that the magnitude of neonatal sepsis was around 50% of the admitted cases to the NICUs [9].

Neonatal sepsis often presents with subtle and nonspecific clinical signs and symptoms that make early identification challenging. As a result, high rates of antibiotics are prescribed empirically, which may contribute to the selection and spread of bacterial strains resistant to antibiotics in a given hospital ward [10]. Despite advances in medical care, neonates remain highly susceptible to infections. Gram-negative bacteria (GNB) represent a substantial proportion of pathogens implicated in neonatal sepsis [11]. The emergence of multidrug-resistant (MDR) strains among GNB further complicates the treatment of neonatal infections and underscores the urgent need for a comprehensive understanding of the antimicrobial susceptibility profile of bacterial strains among neonates with sepsis.

Understanding the distribution of GNB pathogens circulating in neonates admitted to NICU and their antimicrobial susceptibility profile is essential for guiding empiric antibiotics therapy, implementing effective infection control measures, and combating the rising challenges of AMR. There is limited information on the gram-negative neonatal sepsis, bacteriological profile, resistance pattern and treatment outcomes in Ethiopia. The main aim of this study is to assess the distribution of major gram-negative pathogens, their antimicrobial susceptibility profile and treatment outcome among neonates diagnosed with sepsis in two hospitals in Addis Ababa, Ethiopia. The timely, evidence-based distribution of gram-negative sepsis and treatment outcomes will help to strengthen and revitalize existing healthcare policies and develop strategies to reduce the neonatal morbidity and mortality across the country.

## Materials and methods

### Study area and setting

The study was conducted at two public hospitals in Addis Ababa, Ethiopia. Tikur Anbessa Specialized Hospital (TASH) is one of the largest tertiary-level teaching hospitals in Ethiopia, serving as a center of excellence for several clinical service areas across multiple departments with specialty and subspecialty units. The TASH NICU is a 40 bed, level-3 referral unit with approximately 300 monthly admissions. Zewditu Memorial Hospital (ZMH) is a secondary-level facility providing basic specialty-level services and serving as a referral hospital for health centers and three nearby primary hospitals. The ZMH NICU has 31 beds, is a level-2 unit with approximately 175 monthly admissions. Both NICUs are target prevention units (TPUs) supported by a cooperative agreement from the United States Centers for Disease Control and Prevention (CDC) Global Action in Health care Network -Antimicrobial Resistance (GAIHN-AR) program to the Ohio State University (OSU). The GAIHN-AR program strengthen the clinical laboratories capacities and ensure uninterrupted delivery of clinical bacteriology services including blood culture supply and antimicrobial susceptibility testing for its TPUs.

### Study population

All neonates admitted to the NICUs during the study period were considered as a source population. All neonates with a presumptive diagnosis of neonatal sepsis at admission or during hospitalization and having a positive blood culture result during the study period were included in the study. Neonates were excluded if they did not have any blood culture ordered, or if the blood culture obtained had gram-positive organisms or had no growth.

### Study design and period

A retrospective observational study was conducted at TASH and ZMH. The results of all blood cultures submitted between January 2023 to December 2023 were obtained retrospectively from the hospital WHONET microbiological laboratory database. In addition, a retrospective review of medical registry logbook was performed to capture all neonates with admission or in-hospital diagnosis of sepsis.

### Laboratory procedure for identification and reporting of bacteria

One-three milliliter sample of blood was drawn from a fresh venipuncture site and added to a bottle containing 25 mL of tryptone soya broth media. The blood culture bottles were incubated at 35–37°C under aerobic conditions for 24 h and inspected macroscopically daily for seven days for visible evidence of bacterial growth such as hemolysis, turbidity, and gas production. If growth was detected, it was inoculated on to MacConkey, blood and chocolate agar plates. MacConkey agar plates were incubated aerobically at 35–37°C for 24 h and/or sheep blood agar and chocolate agar were incubated at 5% CO_2_ incubator for 24–72 h. The blood culture was considered to be negative, if no bacterial growth was detected on the seventh day. Preliminary bacterial identification was performed based on colony morphology and Gram stain results, which were finally confirmed by using biochemical tests such as indole, citrate utilization, triple sugar iron, urea, mannitol, oxidase, nitrate reduction, lysine decarboxylase, lysine deaminase and motility test were carried out and interpreted as described previously. Muller Hinton agar with 5% sheep blood was used for fastidious organisms. Approximately, three to five pure colony of the test organism was taken by using a sterile wire loop and emulsified in 2 ml of normal saline. McFarland (0.5) was used as a standard to check the 5% turbidity of bacterial suspensions. Then a sterile cotton swab was dipped in to the suspension and squeezed free from excess fluid against the inside wall of the test tube. The test organisms were uniformly seeded on the surface of Muller-Hinton agar for the non-fastidious group and Muller-Hinton agar with 5% sheep blood for the fastidious group and exposed to antibiotics diffusing from the antibiotic-impregnated paper disc into the agar medium and the medium was incubated at 37°C for 24 h.

Antimicrobials tested includes amoxicillin-clavulanate (AMC: 20/10μg), piperacillin/tazobactam (PIP/TAZ: 100/10ug), ceftazidime (CAZ: 30μg), ceftriaxone (CRO: 30μg), cefepime (FEP: 30ug), ertapenem (ETP:10ug), meropenem (MEM: 10ug), amikacin (AMK: 30 μg), gentamicin (GEN: 10μg), ciprofloxacin (CIP: 5μg), and trimethoprim/sulfamethoxazole (SXT: 1.25/23.75μg). The diameter of the zone of inhibition of each antimicrobial disc was measured using calipers to the nearest millimeter and the reading was recorded as susceptible, intermediate and resistant using the Clinical and Laboratory Standard Institute (CLSI) guidelines (CLSI, 2022) [12]. Bacterial culture and antimicrobial susceptibility test (AST) results were submitted electronically to the Ethiopia National WHONET database by each laboratory facility.

### Data analysis and interpretation

Data on the clinical and demographic characteristics of infants including age at admission, sex, clinical diagnosis, length of hospital stay, and treatment outcomes were gathered from medical records and laboratory information on blood culture and antimicrobial susceptibility test results were retrieved from the hospital WHONET database from January, 2024-March,2024. Descriptive analyses such as frequency, proportion, and cross-tabulation were used to summarize the results. To measure the association between the explanatory variable and the outcome of interest, both unadjusted and adjusted odds ratios using a binary logistic regression model were used. p-value <0.05 was considered as a statistically significant association. The collected data were analyzed by using the R statistical software package.

## Ethical consideration

The study was conducted after obtaining ethical clearance from the Ethiopian Public Health Institute review board (EPHI-IRB) with protocol number EPHI-IRB-490–2023. The participants’ data was kept confidential, and the study was conducted under the basic principles of ethics. No individual identifier (fully anonymized) data was collected retrospectively from medical log books.

### Operational definitions

**Suspected Neonatal Sepsis:** Clinical signs and symptoms of infection in the first four weeks of life [13].

**Early onset sepsis (EOS):** Sepsis is diagnosed in the first three days of life.

**Late-onset sepsis (LOS**): Sepsis is diagnosed after three days of birth up to 28 days of life.

**Good outcome:** The attainment of clinical improvement [14].

**Poor outcome:** The attainment of one of the following results: death, self-discharge against medical advice, and referral to another hospital [14].

**Multidrug-resistance (MDR):** Term defined when an isolate is resistant to at least one antibiotic in three or more antimicrobial classes [15].

## Results

**Fig 1:**
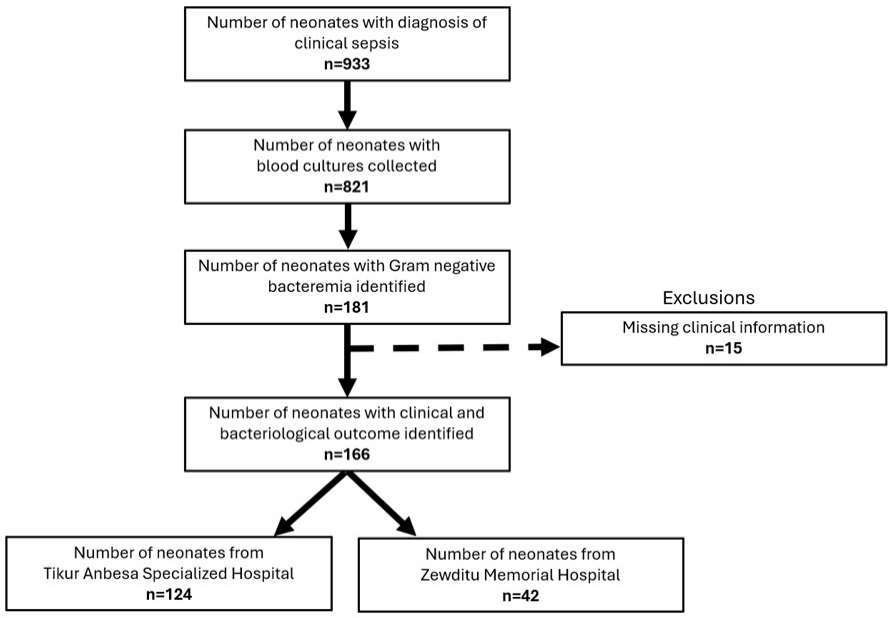
Flowchart for analysis of neonatal sepsis at two hospitals in Addis Ababa, Ethiopia in 2023.

During the one-year study period, a total of 933 newborns were clinically suspected of neonatal sepsis in the NICUs in both hospitals (Fig 1). Among these neonates, 821 had blood culture results, of which 181 (22%) patients had gram-negative bacteremia. Fifteen neonates were excluded due to missing information relating to infant demographics (gender, age at admission, etc.) or lack of treatment outcome and other variables. The final analysis was performed on 166 neonates with 177 GNB isolates.

Summary characteristics of the newborns with neonatal sepsis are provided in Table 1. Among the 166 neonates included in the study, 84 (51%) were female and 97 (58%) had early-onset sepsis. Over half of the neonates were hospitalized for seven days or longer. The median length of hospital stay was nine days with an interquartile range of 16 days. Ninety-two (55%) of the neonates were admitted within 24 hours after birth. Over half of the newborns, 92 (55%) were infected with a MDR-gram-negative pathogen. Seventy-five (45%) of the neonates had poor outcomes observed during their NICU stay while 55% were discharged with good health outcome.

**Table 1:** Demographics, clinical characteristics and outcomes of neonates admitted in TASH and ZMH in January to December 2023.

Variables	Category	Number	Percent
**Sex**	Female	84	51%
	Male	82	49%
**Age at admission**	<=24 hours	92	55%
	>24 hours	74	45%
**Length of Stay**	<7 days	68	41%
	>=7 days	98	59%
**Type of sepsis**	Early Onset Sepsis	148	89%
	Late Onset Sepsis	18	11%
**Multidrug-resistant (MDR) status**	Non-MDR	74	45%
	MDR	92	55%
**Outcome**	Good	91	55%
	Poor	75	45%

TASH: Tikur Anbessa Specialized Hospital; ZMH: Zewditu Memorial Hospital; MDR: multidrug-resistance.

The distribution of bacterial isolates identified from the neonates at both hospitals is shown in Table 2. A total of 177 gram-negative bacteria were isolated from 166 blood specimens collected from neonates (135 from TASH and 42 from ZMH). Eleven patients had polymicrobial gram-negative bacteremia: Four patients with *Acinetobacter spp. and Klebsiella pneumoniae*; three patients with *Acinetobacter spp.* and *Escherichia coli*; two patients with *Enterobacter spp.* and *K. pneumoniae*; one patient with *E. coli* and *K. pneumoniae*; and one patient with *Acinetobacter spp.* and *Klebsiella aerogenes.* The predominant GNB species identified was *Klebsiella spp.* (n = 86; 49%), followed by *Acinetobacter spp.* (n = 38; 21%) and *E. coli* (n = 19; 11%). Fifty-six (41%), 36 (27%), 15 (11%) and 14 (10%) of the isolates identified from TASH were *Klebsiella spp., Acinetobacter spp*. *Enterobact*er spp and *E. coli*, respectively. Whereas 30 (71%), 5 (12%) and 2 (5%) of the isolates identified from ZMH were *Klebsiella spp., E. coli,* and *Acinetobacter spp.,* respectively.

**Table 2:** Distribution of bacterial species isolated from blood specimen of neonates at neonatal intensive care units of TASH and ZMH from January to December 2023.

Species	TASH (n = 135)	ZMH (n = 42)	Overall (N = 177)
*Klebsiella spp.*	56 (41%)	30 (71%)	86 (49%)
*Acinetobacter spp.*	36 (27%)	2 (5%)	38 (21%)
*Escherichia coli*	14 (10%)	5 (12%)	19 (11%)
*Enterobacter spp.*	15 (11%)	1 (2%)	16 (9%)
*Pseudomonas spp.*	6 (4%)	0	6 (3%)
*Citrobacter spp.*	3 (2%)	0	3 (2%)
*Salmonella spp.*	2 (1%)	0	2 (1%)
*Proteus spp.*	2 (1%)	0	2 (1%)
Others ^a^	1 (1%)	4 10%)	5 (3%)

TASH: Tikur Anbessa Specialized Hospital; ZMH: Zewditu Memorial Hospital;

^a^: Non-fermenting gram-negative rods,

*Pantoea agglomerans*

Antimicrobial resistance profile of gram-negative bacteria isolated from TASH, ZMH is shown in Table 3. Among the penicillin class of antibiotics, resistance to amoxicillin/clavulanic acid was observed in 48% (23/48) of the *Klebsiella spp.* while resistance to piperacillin-tazobactam was reported in 26% (9/35) of the isolates. From the cephalosporin groups*, Klebsiella spp.* resistance to ceftriaxone was 89% (68/76) and to cefepime was 89% (56/63). For *Klebsiella spp*., aminoglycosides had high rates of resistance to gentamicin at 75% (60/80) but low rates of resistance to amikacin at 4% (3/70). Resistance of *Klebsiella spp.* to ertapenem and meropenem were 16% (11/68) and 13% (9/69), respectively. The study also found a high rate of resistance among Acinetobacter *spp.:* 64% (16/25) to ceftriaxone, 62% (23/37) to gentamicin, 53% (8/15) piperacillin/tazobactam, and 28% (9/32) to amikacin*.* Among the tested antimicrobials, *E coli* exhibited the highest rate of resistance to trimethoprim/sulfamethoxazole 78% (14/18) followed by ceftriaxone 44% (8/18), gentamicin 33% (6/18) and amoxicillin/clavulanic acid 31% (4/13). The resistance rates of *E. coli* isolate to meropenem and ertapenem were 19% (3/16) and 7% (1/15), respectively. Of note, none of the *Klebsiella* and *E.coli isolates* from ZMH were resistant to carbapenems whereas 23% and 26% of *Klebsiella* and 27% and 10% of *E. coli* isolates from TASH neonates were resistant to meropenem and ertapenem, respectively. Additionally, the resistance rate to amikacin in *E. coli* was 6%. Overall rate of resistance to most of antimicrobials was higher among isolates from TASH compared to those from ZMH.

**Table 3:** Antibiotic resistance profile of the most common gram-negative bacteria isolated from newborns suspected of sepsis at TASH and ZMH from January to December 2023.

Antibiotic name	*Klebsiella spp. % Resistance*			*Escherichia coli % Resistance*			*Acinetobacter spp.% Resistance*		
	TASH	ZMH	Overall	TASH	ZMH	Overall	TASH	ZMH	Overall
AMC	17/22 (77%)	6/26 (23%)	23/48 (48%)	4/8 (50%)	0/5 (0%)	4/13 (31%)	ND	ND	ND
PIP/TAZ	7/18 (39%)	2/17 (12%)	9/35 (26%)	2/9 (22%)	0/4 (0%)	2/13 (15%)	8/15 (53%)	ND	8/15 (53%)
CAZ	44/55 (80%)	20/23 (87%)	64/78 (82%)	4/12 (33%)	0/4 (0%)	4/16 (25%)	24/35 (69%)	2/2 (100%)	26/37 (70%)
CRO	42/48 (88%)	26/28 (93%)	68/76 (89%)	7/13 (54%)	1/5 (20%)	8/18 (44%)	15/24 (63%)	1/1 (100%)	16/25 (64%)
FEP	36/38 (95%)	20/25 (80%)	56/63 (89%)	2/10 (20%)	0/4 (0%)	2/14 (14%)	15/27 (56%)	2/2 (100%)	17/29 (59%)
ETP	11/43 (26%)	0/25 (0%)	11/68 (16%)	1/10 (10%)	0/5 (0%)	1/15 (7%)	ND	ND	ND
MEM	9/40 (23%)	0/29 (0%)	9/69 (13%)	3/11 (27%)	0/5 (0%)	3/16 (19%)	10/35 (29%)	2/2 (100%)	12/37 (32%)
AMK	3/42 (7%)	0/28 (0%)	3/70 (4%)	1/11 (9%)	0/5 (0%)	1/16 (6%)	8/30 (27%)	1/2 (50%)	9/32 (28%)
GEN	38/51 (75%)	22/29 (76%)	60/80 (75%)	5/13 (38%)	1/5 (20%)	6/18 (33%)	21/35 (60%)	2/2 (100%)	23/37 (62%)
CIP	10/39 (26%)	2/28 (7%)	12/67 (18%)	1/7 (14%)	1/5 (20%)	2/12 (17%)	8/28 (29%)	0/2 (0%)	8/30 (27%)
SXT	29/46 (63%)	25/27 (93%)	54/73 (74%)	11/13 (85%)	3/5 (60%)	14/18 (78%)	7/29 (24%)	1/1 (100%)	8/30 (27%)

TASH: Tikur Anbessa Specialized Hospital, ZMH: Zewditu Memorial Hospital, AMC: Amoxicillin/Clavulanic acid, PIP/TAZ: Piperacillin/Tazobactam, CAZ: Ceftazidime, CRO: Ceftriaxone, FEP: Cefepime, ETP: Ertapenem, MEM: Meropenem, AMK: Amikacin, GEN: Gentamicin, CIP: Ciprofloxacin, SXT: Sulfamethoxazole-Trimethoprim, ND: not determined.

Fifty-five percent (92/166) of the neonates had MDR-gram-negative sepsis: 52% of neonates at TASH and 62% neonates at ZMH. Nearly 66% of *Klebsiella spp.,* 32% of *E. coli,* and 47% of *Acinetobacter spp.* were MDRs. *Klebsiella spp.* was the most common MDR pathogens at both hospitals, followed by *Acinetobacter spp..* Among the *Klebsiella* spp. isolates, *K. pneumoniae* accounted for the highest proportion of MDR (51%) followed by *K. oxytoca* (26%). About 44% of the *Acinetobacter spp.* isolates from TASH were MDR compared to 100% of the *Acinetobacter spp.* isolates from ZMH. See Table 4.

**Table 4:** Frequency distribution of multidrug-resistance organisms at TASH and ZMH from January to December 2023.

Site	# and % of Total MDR	Number and (%) of MDR				
		*Klebsiella spp*.	*Escherichia coli*	*Acinetobacter spp.*	*Enterobacter spp.*	Others
TASH	65/124 (52.4%)	37/56 (66%)	5/14 (36%)	16/36 (44%)	5/15 (33%)	5/14 (36%)
ZMH	26/42 (62%)	20/30 (67%)	1/5 (20%)	2/2 (100%)	1/1 (100%)	2/4 (50%)
Total	92/166 (55%)	57/86 (66%)	6/19 (32%)	18/38 (47%)	6/16 (38%)	8/18 (44%)

MDR: Multidrug-resistant; TASH: Tikur Anbessa Specialized Hospital; ZMH: Zewditu Memorial Hospital.

Table 5 summarizes the results from binary logistic regression, which shows the associations between poor health outcomes inclusive of both demographic and clinical characteristics. Among the factors included in the study, the length of hospital stays, and the antibiotic resistance pattern of the bacterial infection, were statistically significant in the health outcome of neonates. Neonates with MDR infections had a 5-fold increased probability of experiencing poor health outcomes compared to those with non-MDR bacterial infections (AOR: 5.2 95% CI (2.588–11.110). Newborns who stayed less than seven days, compared to those who spent seven or more days in the hospital were four times (AOR: 4.16 95% CI (2.0–9.0) more likely to experience poor health outcomes. While not statistically significant, male newborns, early admitted infants within first 24 hours of birth, and infants with late onset sepsis were less likely to have poor health outcomes.

**Table 5:** Analysis of factors associated with the poor health outcome of sepsis among neonates at TASH and ZMH from January to December 2023.

Patient characteristics	Category	% of poor Outcome	COR (95% CI)	AOR (95% CI)
Sex	Male (n = 82)	43%	0.819 (0.44 - 1.51)	0.720 (0.35 - 1.45)
	Female (n = 84)	48%	Ref	Ref
Age at admission	<=24 hrs. (n = 92)	44%	0.857 (0.44 - 1.51)	0.756 (0.34- 1.62)
	>24 hrs. (n = 74)	47%	Ref	Ref
Length of Stay	<7days (n = 68)	62%	3.22 (1.69-6.25)	4.16 (2.0 - 9.09)
	≥7days (n = 98)	34%	Ref.	Ref.
Sepsis type	LOS (n = 18)	11%	0.572 (0.19 - 1.55)	0.688(0.18 - 2.38)
	EOS (n = 148)	89%	Ref	Ref
Multidrug-resistant status	Non MDR (n = 78)	27%	Ref	Ref
	MDR (n = 88)	61%	4.31 (2.25 - 8.46)	5.231 (2.59-11.0)

COR: crude odds ratio, AOR: adjusted odds ratio, CI: confidence interval, LOS: length of stay, EOS: Early onset sepsis, Ref: Reference

## Discussion

Antimicrobial resistance has been increasing globally and poses a public health threat, especially in low middle income countries (LMICs). Targeted antimicrobial therapy is the basis for improving clinical outcomes and survival rates in neonatal sepsis. However, the AMR pattern and bacteriological profile of the pathogens causing newborn sepsis in low-resource settings, especially gram-negative bacteremia have not been extensively studied. Despite the limited sensitivity of blood cultures, due to low volume collected in neonates and the frequent administration of empiric antibiotics before specimen collection in the NICU, blood cultures remain the gold standard for diagnosing newborn sepsis [16,17].

One hundred and forty-eight (89%) neonates involved in the current study were admitted with the diagnose of early onset neonatal sepsis (EONS). A similar study conducted in Northern part of Ethiopia reported that 82.5% of neonates admitted to NICU had early neonatal sepsis [17]. On the other hand, studies conducted in India and Egypt showed that the EONS was lower at 59% and 42% respectively [18,19]. These disparities can be explained by the fact that the prevalence of neonatal sepsis varies geographically and depends on the quality of healthcare service deliveries at the facility moreover difference in infection prevention and control practices in the health care center. The median length of stay in the NICU in the this study was nine days, whereas, in the previous multi-center study conducted in Ethiopia showed that the median stay for the neonate was seven days [13]. This may be due to multiple factors such as severity of sepsis and health service delivery. In our study, the majority of the neonates admitted to both hospitals were infected with MDR-GNB. Higer rate of sepsis with MDR-GNB has also been reported in a previous study in Ethiopia [19].

The etiology of newborn sepsis is diverse and varies from country and region. For instance, before the introduction of intrapartum antibiotic prophylaxis, *S. agalactiae* was one of the primary causes of neonatal sepsis in the United States [20]. But in Ethiopia, this bacterium is seldom isolated from infants who may have sepsis. In our study, the most frequently identified bacteria from both hospitals were *Klebsiella spp.* (49%), *Acinetobacter spp.* (21%), and *E. coli* (11%). St. Paul Millenium Hospital, a tertiary hospital, in Addis Ababa, Ethiopia, reported similar *Klebsiell*a *spp.* (44%) and *E. coli (23%*) prevalence rate [21]. But Ayder Tertiary Hospital, in northern Ethiopia, reported lower rates for *Klebsiella spp*. (35%) and *E.coli (6%*) [22]. The incidence rates could vary by locality, area of catchment the hospital services, as well as the type of referral facility.

*Klebsiella spp*. showed a significant level of antibiotic resistance to cephalosporins, but was mostly susceptible to other classes of antibiotics such as ciprofloxacin, amikacin, and carbapenems. This finding outcome is consistent with previous study reports from Ethiopia [23], Egypt [24] and Zambia [25]. This may be due to wide availability and use of cephalosporins as first line empiric antimicrobials in the NICUs.

The bacterial resistance for carbapenems varies across the hospital in different regions, our high overall susceptibility for meropenem (87%) and ertapenem (84%) is consistent with other studies in Ethiopia, Zambia, and Egypt [25, 26, 27] but lower than a study report from Nepal [28]. The pooled analysis done in Africa showed that the prevalence of carbapenem resistance was 30.4% and the pooled gram-negative resistance to imipenem and meropenem was 35.6% and 34.4% respectively [29]. Identification of carbapenem resistance is concerning in LMICs due to the lack of alternative therapeutic agents and the inability to even test for antimicrobial susceptibility to the newer antimicrobial agents. Of the *Acinetobacter spp.* reported, 32% were resistant to meropenem, which is similar to the study report done by Tadesse *et al* in Ethiopia [23].

According to the study, there was a 55% prevalence of multidrug-resistance organisms (MDROs) in both institutions. The main MDRO was *Klebsiella spp*. A similar study reported, that the prevalence of MDROs in neonatal sepsis was 65% and 84% in northern and central Ethiopia [23,31]. A higher prevalence of MDROs related sepsis is observed in Ethiopia and other sub-Saharan African nations [10,32]. Among *Klebsiella* spp. *K. pneumoniae* is accounted for higher proportion of MDRs (51%) followed by *K. oxytoca* (26%). A similar study conducted at Felge Hiwot Specialized Hospital in northern Ethiopia showed that 48.6% of the samples from the blood cultures grew an extended spectrum beta lactamase (ESBL) producing *K. pneumoniae* [30]. Another study performed in Ethiopia reported that the pooled proportion of estimated ESBL producing *K. pneumonia*e was 61.8% [31]. While a study done in Iran reported that the prevalence of pooled nosocomial MDR *K. pneumoniae* was 55% [33]. This variation can be due to differences in population size, health care setup, antimicrobial availabilities, and prescription practices. Moreover, extensive and unregulated over use of antibiotics, invasive procedures such as catheterization, poor hand hygiene practices by health care workers and environmental factors, all contribute to the evolving of MDROs in the NICU [34].

Furthermore, our study also revealed that newborns with MDR infection had five times higher [AOR, 5.23 CI (2.59, 11.11)] poor treatment outcomes during their hospital stays compared to newborns without MDR infections. This result is consistent with a previous study report from Myanmar and Pakistan [35,36]. This might be explained by MDROs infection association with severe diseases and limited therapeutic antibiotic possibilities to treat the MDRO infections in neonates compared with adult population [37]. Poor infection prevention and control (IPC) practices and long-term antibiotic exposure were reported to be risk factors for MDR [34]. IPC strategies along with antimicrobial stewardship programs to routinely evaluate the patient’s clinical progress, dose, duration, and reason for antibiotic continuation should be implemented to improve outcome and decrease AMR in the NICUs.

On the other hand, newborns who stayed less than seven days, compared to those who spent seven or more days in the hospital were four times (AOR: 4.16, 95% CI (2.0–9.0) more likely to experience poor health outcomes. A similar study in Pakistan reported that neonate who had longer hospital stay were 24% less likely experienced death in the hospital [35]. This finding could be explained by the fact that patients with severe illnesses died earlier than those with mild to moderate infections. Additionally, a multi-center study done in public hospital in Addis Ababa showed that the majority of deaths occurred within the first five days of births even though these deaths were not solely contributed by antimicrobial resistance [38]. Lastly, due to secondary data source limitation, we did not have information on maternal factors, prior antibiotic use, birth weight, other clinical and socioeconomic factors that may have contributed to sepsis and clinical outcome.

## Conclusion

Most of the neonates were admitted with the diagnosis of early-onset neonatal sepsis in both NICUs. Among the common gram-negative bacterial bloodstream infections, *Klebsiella spp*. was the most common pathogen identified at both hospitals. More than half the neonatal sepsis was caused by MDROs and was significantly associated with poor treatment outcomes compared with non-MDROs. The higher rate of MDROs and high resistance to the commonly prescribed antimicrobials is alarming. Some key measures are needed to decrease emergence and transmission of MDROs. Stringent IPC measures, strong surveillance and antimicrobial stewardship programs are recommended to curb the clinical impact of MDR pathogens.

## Supporting information

S1 Data Patient characteristic. (XLSX)

S2 Data Laboratory data. (XLSX)

S3 Data Data (NICU - AMR Data set). (XLSX)

## Abbreviation

EONS: Early onset neonatal sepsis
GNB: Gram-negative bacteria
LOS: Late onset sepsis
MDR: Multidrug-resistant
MDRO: Multidrug-resistance organism
NICU: Neonatal intensive care unit
TASH: Tikur Anbessa Specialized hospital
TPU: target prevention unit
ZMH: Zewditu Memorial Hospital
